# The Effect of Stimulus Duration and Motor Response in Hemispatial Neglect during a Visual Search Task

**DOI:** 10.1371/journal.pone.0037369

**Published:** 2012-05-25

**Authors:** Laura M. Jelsone-Swain, David V. Smith, Gordon C. Baylis

**Affiliations:** Department of Psychology, University of South Carolina, Columbia, South Carolina, United States of America; Katholieke Universiteit Leuven, Belgium

## Abstract

Patients with hemispatial neglect exhibit a myriad of profound deficits. A hallmark of this syndrome is the patients' absence of awareness of items located in their contralesional space. Many studies, however, have demonstrated that neglect patients exhibit some level of processing of these neglected items. It has been suggested that unconscious processing of neglected information may manifest as a fast denial. This theory of fast denial proposes that neglected stimuli are detected in the same way as non-neglected stimuli, but without overt awareness. We evaluated the fast denial theory by conducting two separate visual search task experiments, each differing by the duration of stimulus presentation. Specifically, in Experiment 1 each stimulus remained in the participants' visual field until a response was made. In Experiment 2 each stimulus was presented for only a brief duration. We further evaluated the fast denial theory by comparing verbal to motor task responses in each experiment. Overall, our results from both experiments and tasks showed no evidence for the presence of implicit knowledge of neglected stimuli. Instead, patients with neglect responded the same when they neglected stimuli as when they correctly reported stimulus absence. These findings thus cast doubt on the concept of the fast denial theory and its consequent implications for non-conscious processing. Importantly, our study demonstrated that the only behavior affected was during conscious detection of ipsilesional stimuli. Specifically, patients were slower to detect stimuli in Experiment 1 compared to Experiment 2, suggesting a duration effect occurred during conscious processing of information. Additionally, reaction time and accuracy were similar when reporting verbally versus motorically. These results provide new insights into the perceptual deficits associated with neglect and further support other work that falsifies the fast denial account of non-conscious processing in hemispatial visual neglect.

## Introduction

Hemispatial visual neglect is a syndrome of attention deficit that frequently occurs after unilateral brain damage, such as from stroke. Patients with neglect are unaware of, or unresponsive to, information in the side opposite their damage (referred to as the contralesional side) [1]. For example, they may shave only one side of their face or color only the ipsilesional side of a picture. Despite the obvious behavioral impairments of visual neglect, patients with this disorder often display marked anosognosia [2] (i.e., they appear to lack awareness of their inattention).

Although people with neglect may lack explicit awareness of information in their contralesional space, it is well established that unconscious processing of this visual field can take place. This phenomenon has been repeatedly demonstrated across different modalities and behavioral functions [3]–[14]. For example, one highly cited study described a patient with left-sided neglect who was shown two drawings of the same house, however the left side of one of these houses was in flames [10]. When asked to choose the house she would prefer to live in, she consistently chose the non-burning house while claiming that both were identical. Even though such examples like this are common, the mechanistic underpinnings behind unconscious processing of neglected information are still under debate.

One theory was proposed to explain the phenomenon of unconscious processing after a hemispatial neglect patient appeared to rapidly deny contralesional stimuli during a visual search task [15]. This patient was faster to correctly report the presence of an ipsilesional target than to report (true) target absence (presumably reflecting the fact that a stimulus onset terminates the search). Paradoxically, the patient was also rapid at neglecting targets presented in the contralesional field. The authors concluded that this behavior represented a form of residual awareness to these neglected stimuli. That is, the patient's behavior (reaction time) was the same for stimuli being present regardless of the actual output (“yes” or “no”). According to the authors, the processes involved in detecting these stimuli were intact, but the stimuli never reached conscious recognition. Thus, this patient's rapid and incorrect reply was a “fast denial” of unconsciously processed information.

Laeng and colleagues examined the fast denial theory in a single patient case study [16]. The results of this study did not support the fast denial theory because their patient was faster to neglect stimuli than to detect them. A major difference between the studies conducted by Mijović-Prelec [15] and Laeng [16] was that one required a verbal response and the other a motoric button-press. Therefore, interpretation of differences from the results between these two case studies is limited given that the measure of reaction time is based on different methods of reporting, and both studies examined behavior from only one person.

Motor and higher level visual systems are directly influenced by each other. For example, visual feedback is necessary to guide movements such as reaching. Mattingley et al. [17] demonstrated a relationship between motor planning and visual awareness in neglect patients. It was concluded that this relationship stems from functioning of the inferior parietal lobe rather than from the frontal lobe, as was previously thought. It can be inferred from this study that dual functioning of the inferior parietal lobe, an area commonly damaged in neglect patients [18]–[20], might result in altered visual search abilities when a motor response is required. Of course, lateralized motor responses by neglect patients during visual search tasks and exploration can be influenced by visual information [21], [22], [23]. However, it is not known how motor responses are affected when compared to verbal responses during visual search processes if non-lateralized motor movements are required.

The underlying purpose of our study was to examine the theory of fast denial in a group of patients with neglect. We tested this theory with two different manipulations. First, we aimed to compare verbal and motor responses during a visual search task. To the best of our knowledge, no study to date has directly examined neglect behavior between these two response types with a neglect patient group. In addition to addressing response modality, as this may have contributed to differences seen between previous studies [15], [16], motor and verbal responses were compared to test for the possible interchangeability between these two methods in future studies.

Our second aim was to examine duration effects in a visual search task. Therefore we compared responses during two visual search tasks when the target either remained in the visual field or was displayed for only several hundred milliseconds. Many visual search tasks display target stimuli for an unlimited amount of time in the visual field. However, we often experience brief intervals of information under normal conditions, and this brief presentation of information may affect unconscious processing differently in patients with neglect.

We conducted two experiments to test our aims. The main difference between experiments was that each stimulus in Experiment 1 remained on the computer screen until a response was made and in Experiment 2 each stimulus was displayed briefly. Also, the paradigm in Experiment 1 was similar to that designed by Mijović-Prelec et al. [15] in attempt to induce fast-denial responses, whereas the template of the visual search task was simplified in Experiment 2. All other factors remained the same, and a verbal and motor task was included in each experiment. Verbal and motor responding was not included within the same task to eliminate a possible interaction effect [23]. It was hypothesized that reaction times to neglected stimuli would be longer in Experiment 1 given the unlimited amount of time to process each stimulus, assuming that unconscious processing is taking place. It was also hypothesized that reaction times during the motor modality would be longer due to the hand-motor coordination needed to press the target button, and because of the additional component of having to associate the chosen response (yes or no) with the appropriate button.

## Methods

### Ethics Statement

The Institutional Review Board at the University of South Carolina approved all procedures, and all participants provided written informed consent prior to their participation in any of the assessments or experiments.

### 2.1. Participants

A total of 28 participants were recruited in our study. All participants were without any visual impairment that could have affected their performance during the experiment, including hemianopia. Participants enrolled in the study on a full voluntary basis, with no monetary compensation for their time. Patients with neglect and control patients with parietal damage without neglect were recruited from Health South Rehabilitation Center (Columbia, South Carolina). Healthy control participants were recruited from the general community.

#### 2.1.1. Participants with Hemispatial Neglect

Twelve patients with hemispatial neglect resulting from stroke were recruited for this study. These patients were without speech or ipsilateral motor impairment that would have impacted their reaction time in this study. Seven patients completed Experiment 1, of which three completed both the verbal and the motor task, resulting in five completed data sets for the motor and verbal task each (*M*_age-motor_ = 65.4, *M*_age-verbal_ = 70.4). Eight patients with neglect participated in Experiment 2 (*M_age_*_-motor_ = 63.8, *M*_age-verbal_ = 69.8). Two patients completed both tasks, resulting in five data sets per task. Three patients were only able to complete one block in the motor task. All patients performed the experiment in their hospital rooms, except for patient #8 who was recruited as an outpatient participant. All patients were assessed for neglect symptoms using the diagnosis criteria discussed below. See Table 1 for neglect patient demographic information.

**Table 1:** Demographic information, tasks performed, and percent of contralesional stimuli neglected in each task for all participants with visual neglect, in both Experiments 1 and 2.

ID#	SEX	AGE	LESION LOCATION	LESION DURATION	DOMINANT HAND	TASKS COMPLETED	% MISS RATE
**1**	M	71	R basal ganglia	8 days	R	Exp 1 motor	96
						Exp 1 verbal	97
						Exp 2 verbal	100
**2**	M	62	R temporal	12 years	R	Exp 1 motor	37
						Exp 1 verbal	57
						Exp 2 motor	37
**3**	F	75	R temporo-parieto-occipital junction and thalamus	5 months	R	Exp 1: motor	39
						Exp 1 verbal	42
**4**	F	81	R middle cerebral artery	22 days	R	Exp 1: motor	90
**5**	M	38	R basal ganglia	18 days	R	Exp 1: motor	100
**6**	M	62	R basal ganglia – thalamus	40 days	R	Exp 1: verbal	38
						EXP 2 verbal	32
**7**	F	76	R posterior parietal	3 years; 25 days	R	Exp 1: verbal	38
**8**	M	80	L posterior parietal	19 months	R	Exp 2: motor	98
						Exp 2 verbal	100
**9**	M	54	R basal ganglia and thalamus	2 months	R	Exp 2: motor	100
						Exp 2 verbal	83
**10**	M	72	R temporal-parietal	19 days	R	Exp 2: motor	100
**11**	F	57	R posterior parietal -thalamus	16 days	R	Exp 2: motor	54
**12**	M	82	R frontal-parietal	12 days	L	Exp 2: verbal	72

Age is in years, lesion duration is between the time of stroke and testing session, and dominant hand is self-reported.

Lesion location is based on neurological medical report at time of admittance into Health-South Rehabilitation Center.

Patient 7 suffered from first stroke three years before but was readmitted to hospital 25 days prior to participation after suffering a second stroke.

#### 2.1.2. Participants with Parietal Lesions Without Neglect

This control group included 11 stroke patients with lesions in their parietal lobe, without history or current symptoms of neglect. This group was chosen as a control because parietal injury often leads to hemispatial neglect [24]. In Experiment 1, seven patients total participated (M_age-motor_ = 58.6, *M*_age-verbal_ = 65.75). Three of these participated in both the motor and verbal task, resulting in five data sets per task. In Experiment 2, seven patients total participated (*M*_age-motor_ = 64.8, *M*_age-verbal_ = 56.66). Two of these completed both motor and verbal tasks, and one participant in each task was only able to complete one block. Overall there were five data sets created in the motor task and four in the verbal task in Experiment 2. Three patients had right parietal damage in each experiment and all were right handed. All patients performed the experiment while in their hospital rooms.

#### 2.1.3. Healthy Controls

Five healthy volunteers without any history of brain damage or head trauma were recruited. Five people participated in each task in Experiment 1 (*M*_age_ = 64.6). Five people participated in Experiment 2; three completed both tasks resulting in four participants per task (*M*_age-motor_ = 64.25, *M*_age-verbal_ = 65.25). All participants were right handed.

### 2.2. Neglect Diagnosis Criteria

All participants were given a battery of three tests in order to assess hemispatial visual neglect behavior. 1) Clinical confrontation test: The experimenter stood in front of the participant's midline, held their index fingers upright in each visual field, and wiggled their finger one at a time at four different visual angles. Each patient was asked to keep their eyes fixated at centerline and to report what side of their peripheral space they detected movement. A patient was considered to have neglect when they missed more than 30% of the stimuli presented in the contralesional hemispace. 2) Line-bisection test: Patients were asked to bisect a nine-inch horizontal line (on paper). This paper was positioned central to the patient's midline. At least a 10% deviation toward the ipsilesional side (from the middle at 0%) suggested visual neglect. 3) Clock-drawing test: Patients were asked to manually write the numbers inside a circle (6″ diameter) that was described to them as a “clock-face”. A spatial bias toward the ipsilesional side indicated possible neglect, such that the majority of numbers were located within this space of the paper. All patients who were considered to have visual neglect and participated in the current study met at least two of these three criteria. No control participant displayed any symptoms suggestive of neglect behavior.

### 2.3. Protocol and Materials

#### 2.3.1. Stimuli and Apparatus

A Cornea TFT LCD flat-screen monitor (screen size: 14×11 inches) was used to display each visual search task. The experimenter controlled the search task from a Hewlett Packard laptop that was connected to the monitor. The monitor was placed central to the patient's midline, approximately three feet from their body. The search tasks were programmed in E-Prime, version 1.1 (Psychology Software Tools, INC).

#### 2.3.2. Experiment 1 Paradigm

**Figure 1:**
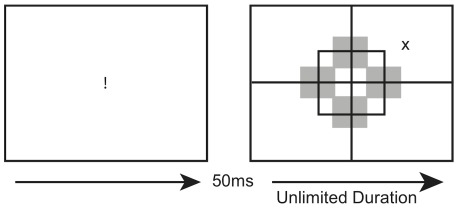
Example of a single possible trial in Experiment 1 when a stimulus appeared in the upper right quadrant (the ipsilesional side for a patient with right hemisphere damage).

The paradigm of Experiment 1 closely replicated that of Mijović-Prelec, et al. [17] (see Figure 1). Each block contained 72 pseudo-randomly assigned (without replacement) trials, in which one or two blocks were completed for each task. The target, a black “X” (5×3 cm), appeared a third of the time in the left and right visual fields each, and a third of the time it did not appear. When present, the target appeared equally in a fixed location in the center of any one of the four quadrants. This stimulus followed 50 ms after fixation (exclamation point, 4×1 cm).

#### 2.3.3. Experiment 2 Paradigm

**Figure 2:**
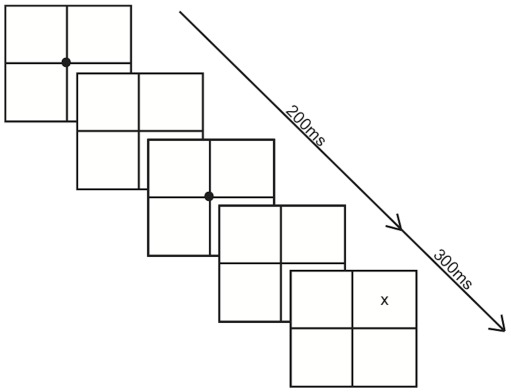
Example of a single possible trial in Experiment 2 when a stimulus appeared in the upper right quadrant (the ipsilesional side for a patient with right hemisphere damage).

There were only minor differences in the paradigm of Experiment 2 compared to Experiment 1. First, the stimulus was only presented for a fixed duration. Second, the template design of the paradigm was simplified (see Figure 2) to help produce a pop-out effect. A circle fixation cue (3 cm in diameter) in the center of the screen prompted each trial. This circle flashed twice (50 ms duration each) and 50 ms later either the target (a 3×5 cm black X) would or would not appear (200 ms onset from trial initiation). The target appeared two-thirds of the time out of 72 trials (per block), and occurred equally in either the right or left visual field. All stimuli were presented pseudo randomly without replacement. Baylis et al [24] demonstrated that 400 ms was the average calibrated stimulus duration needed for patients with extinction, a disorder similar to neglect, to be capable of performing above chance in detecting flashing stimuli. Therefore we chose to display each stimulus for 300 milliseconds as the baseline for each participant during the practice session. This stimulus duration was checked to verify that detection of targets (in the ipsilesional field for neglect participants) was above chance in the neglect patient group and above 90% in the control groups. This duration time was too fast for neglect patient #12 and therefore adjusted to 700 ms.

#### 2.3.4. Verbal Task

An E-Prime compatible microphone connected to a serial subject response box (Psychology Software Tools, INC.) was used. Reaction time for each trial was recorded as the time between stimulus onset and the participant's response. Voice activation into the microphone terminated each trial. Each participant's response was manually entered into the computer by the experimenter, who then started the next trial. Verbal responses were all unambiguous.

#### 2.3.5. Motor Task

Two round plastic buttons (“jelly-bean” switches), one red and one green (34 cm in diameter), were secured on top of a portable “lap desk”, which sat comfortably on the participant's lap. The jelly-bean buttons were placed vertically in front of the participant on their ipsilesional side to ensure no lateral confound was induced [25], [26]. These buttons remained in-line with each participant's ipsilesional arm, therefore not requiring the participant to make a lateral movement with their arm or hand. Both buttons were connected to the HP laptop by an E-Prime compatible serial response box (Psychology Software Tools, INC.). Reaction times were recorded as the time between stimulus onset and button press, and E-Prime recorded which button was pressed for each trial. Trials were terminated when either button was pressed. Like in the verbal task, the experimenter controlled the onset of each new trial.

#### 2.3.6. Procedure

Participants were told to respond to whether they saw the stimulus, that the stimulus was an X, and that it would either appear in the center of any of the four quadrants or not at all. Instructions were to verbally or motorically report the presence or absence of the stimulus in each trial, and to make their responses as quickly and accurately as possible. Each participant was given a 12-trial (equal number of all stimulus conditions) practice session before they began the experiment.

In the verbal task, participants held the microphone in a fixed position near their mouth throughout the session and were asked to speak either “yes” or “no” into the microphone for each trial. In the motor task, participants were instructed that the green button symbolized “yes” and that the red button symbolized “no.” Each participant was familiarized with the buttons to ensure they comprehended this instruction. The motor and verbal tasks were counterbalanced across participants. Button position (red vs. green in the upper position) was also counterbalanced across both participants and block sessions (for those who completed two blocks) in the motor task.

#### 2.3.7. Statistical Analysis

It was our goal to compare motor and verbal responses in each experiment. Additionally, our goals were to measure neglect behavior to timed and untimed stimulus presentations during verbal and motor responses, and to assess if these measurements fit a fast denial model.

We used a linear mixed effects model to examine the effects of task (motor and verbal) and stimulus type (contralateral, ipsilateral, and absent stimuli conditions) on the outcome variable reaction time in each experiment. In the neglect patient group the contralateral stimulus condition was specific to targets presented in the contralesional space that were neglected and the ipsilateral condition was specific to correctly reporting stimulus presence in the ipsilesional space. Correctly responding to contralesional stimuli in the neglect group was not part of the *a priori* hypothesis, therefore was not included in the analyses. In the control groups, the contralateral stimulus condition was measured as the correct responses to stimulus presence. Therefore, a single statistical model combining all groups could not be performed. The left side was considered the healthy control group's contralateral side given that all participants in this group were right handed.

The linear mixed effects model was conducted in Experiments 1 and 2 for each group, in order to account for correlated data across tasks that incorporated a mixture of independent and repeated subject samples. Task (verbal and motor) and stimulus type (contra, ipsi, and no) were included as the fixed effects measures. An interaction between task and stimulus was also included in the model but because no interactions were significant they will not be discussed further. The intercept was included as the random effect measure and stimulus type was also included as a repeated measures variable. Variance components heterogeneous compound symmetry was chosen for the covariance structure and the restricted maximum likelihood estimation was used.

The account of a fast denial involves reacting to neglected and detected stimuli equally fast. According to this theory, this is because the neglected stimuli are detected in the same manner as the non-neglected stimuli, however this detection fails to reach conscious awareness. Because we were specifically interested in comparing reaction times to neglected and detected stimuli, we conducted paired sample t-tests for all stimulus condition comparisons within each experiment and task.

Exploration of the raw reaction times (RT) of each participant in each condition indicated that RTs were positively skewed. Therefore, logarithmic (base 10) transformations of individual raw RTs in each condition per each participant were performed. All statistical tests measuring reaction time were analyzed using these transformed means. To allow for interpretation of the data, raw reaction times of the neglect group for each condition are presented at the individual level (see Tables 2 & 3). The raw data are shown using the trimmed means (top and bottom 2.5% removed), which is a robust measure of central tendency. All statistical results were derived from the statistical package SPSS, version 19.0.

**Table 2:** Trimmed means (top and bottom 2.5% removed) showing robust center of raw reaction times (ms) and standard errors (from non-trimmed raw data) for individual neglect patients and control groups in Experiment 1.

PARTICIPANTS	MOTOR TASK			VERBAL TASK		
Neglect Patients	CONTRA	IPSI	NO	CONTRA	IPSI	NO
**1**						
Mean	1769.135	1211.565	1535.190	1703.994	943.997	2091.050
SE	355.048	128.160	353.587	370.576	41.819	424.170
**2**						
Mean	1343.537	1133.934	2337.262	925.856	793.566	1215.296
SE	156.924	121.897	329.152	106.248	23.875	103.122
**3**						
Mean	3008.924	1753.527	3606.605	1785.208	1115.905	2502.639
SE	621.197	479.420	420.251	249.041	69.784	387.940
**4**						
Mean	4913.120	2717.630	5591.963	NA	NA	NA
SE	384.152	260.881	535.240	NA	NA	NA
**5**						
Mean	5277.773	1230.329	4395.593	NA	NA	NA
SE	695.645	34.885	487.075	NA	NA	NA
**6**						
Mean	NA	NA	NA	4494.778	1891.622	3697.188
SE	NA	NA	NA	628.803	343.630	317.200
**7**						
Mean	NA	NA	NA	5803.852	4215.931	5956.571
SE	NA	NA	NA	1272.814	720.356	689.287

GROUPS	CONTRA	IPSI	NO	CONTRA	IPSI	NO
**Neglect Patients**						
Mean	3262.500	1609.400	3493.323	2942.737	1792.204	3092.549
SE	442.593	205.048	425.061	525.496	239.893	384.344
**Parietal Controls**						
Mean	1087.605	978.351	1456.076	1064.739	894.559	1684.411
SE	73.558	48.104	135.852	72.992	57.474	183.062
**Healthy Controls**						
Mean	742.707	753.757	863.273	670.270	661.677	713.022
SE	28.118	29.854	35.004	18.647	18.832	15.086

CONTRA (in the neglect group) = misses of contralesional targets; CONTRA (in the control groups) = hits of contralesional/lateral targets; IPSI = hits of ipsilesional/lateral targets; NO = correct response to target absence.

**Table 3:** Trimmed means (top and bottom 2.5% removed) showing robust center of raw reaction times (ms) and standard errors (from non-trimmed raw data) for individual neglect patients and control groups in Experiment 2.

PARTICIPANTS	MOTOR TASK			VERBAL TASK		
Neglect Patients	CONTRA	IPSI	NO	CONTRA	IPSI	NO
**2**						
Mean	2934.592	980.015	3438.520	NA	NA	NA
SE	499.442	38.301	520.577	NA	NA	NA
**8**						
Mean	3259.767	843.164	3007.937	1011.849	652.750	897.184
SE	433.989	168.764	581.518	229.861	140.402	284.673
**9**						
Mean	1692.303	519.359	1567.687	4729.400	709.161	5198.750
SE	210.080	32.373	284.576	461.266	59.660	357.408
**10**						
Mean	3979.310	993.357	6363.340	NA	NA	NA
SE	835.158	184.113	1293.320	NA	NA	NA
**11**						
Mean	4843.680	1002.444	4621.804	NA	NA	NA
SE	760.965	84.061	784.236	NA	NA	NA
**12**						
Mean	NA	NA	NA	4739.556	1229.004	4372.722
SE	NA	NA	NA	532.599	101.274	356.090
**1**						
Mean	NA	NA	NA	13033.170	816.956	8428.601
SE	NA	NA	NA	2483.734	54.245	1467.167
**6**						
Mean	NA	NA	NA	7192.278	1336.133	8744.317
SE	NA	NA	NA	954.701	154.672	811.444

GROUPS	CONTRA	IPSI	NO	CONTRA	IPSI	NO
**Neglect Patients**						
Mean	3341.930	867.668	3799.858	6141.252	948.800	5528.314
SE	547.927	101.522	692.845	932.432	102.051	655.356
**Parietal Controls**						
Mean	858.373	840.345	1848.351	680.813	640.078	1257.415
SE	52.516	44.438	284.024	42.896	44.190	121.804
**Healthy Controls**						
Mean	666.188	675.952	922.055	673.226	645.031	989.698
SE	24.010	25.545	118.953	15.804	19.613	87.503

CONTRA (in the neglect group) = misses of contralesional targets; CONTRA (in the control groups) = hits of contralesional/lateral targets; IPSI = hits of ipsilesional/lateral targets; NO = correct response to target absence.

Neglect patients 2, 10, and 11 were only able to complete one block of the motor task.

## Results

### 3.1. Accuracy: No Differences in Neglecting Between Motor and Verbal Tasks

A primary goal of our study was to examine if performance during a visual search task is affected by response modality or the duration of stimulus presentation. Looking specifically at responses made to stimuli presented in the contralesional field, patients with neglect consistently neglected these stimuli regardless of how they responded. The average miss rate in Experiment 1 for this group was 72.2% (SE = 14.1) and 54.3% (SE = 11.4) in the motor and verbal tasks, respectively. In Experiment 2, miss rate averages in the motor and verbal tasks were 77.4% (SE = 12.5) and 77.8% (SE = 13.5), respectively. Miss rate percentages of contralesional stimuli per neglect participant are presented in Table 1. All patients with neglect except for patient 10 correctly identified ipsilesional stimuli at least 80% of the time and correctly reported stimulus absence at least 90% of the time. Patient 10, who had severe neglect, missed stimuli in their ipsilesional field 62% of the time in Experiment 2 during the motor task. All control participants correctly responded with over 90% accuracy for all three stimuli conditions.

Non-parametric independent sample tests (Mann-Whitney) were performed to compare task and experiment neglect rates (of contralesional stimuli) after removal of repeated subjects. In order to conduct these analyses with independent samples, tasks were compared including individuals in both experiments, and experiments were compared including individuals in both tasks. There was no difference in miss rates between task (U = 7, Z(11) = −1.47, *p* = .14) or experiment (U = 9, Z(9) = −.25, *p* = .80).

### 3.2. Reaction Time

#### 3.2.1. Participants with Neglect Were Equally Slow to Neglected and Absent Stimuli

Again our goals in this study were to test if hemispatial visual neglect is affected by methods of response and to further evaluate the fast denial theory. Therefore we examined the speed of reaction between tasks (verbal and motor) and stimulus type (contralesional misses, ipsilesional hits, correct target absence responses) in both experiments using a linear mixed effects model. There was a significant effect of stimulus type in both Experiments 1 and 2, *F*(2, 8.58) = 38.49, *p*<.001, *F*(2, 8.40) = 27.15, *p*<.001, respectively. The motor task was slower in Experiment 1, *F*(1, 7.34) = 7.64, *p* = .03, but there was no task effect in Experiment 2, *F*(1, 7.88) = .73, *p* = .42.

Taking a closer look at the significant task effect in Experiment 1, it appears that this result may have been driven by the three subjects who participated in both tasks. Overall the group means are very similar between tasks (see Table 2), however participants 1, 2, and 3 were slower in the motor task. These participants contributed twice the marginal percentage in the mixed model compared to those who only participated in one task. Therefore a third linear mixed model was conducted to examine reaction times between the motor and verbal task after collapsing participants between experiments (removing repeated subjects who completed the same task; no subjects were repeated within the same group). This model yielded null results between tasks, *F*(1, 6.94) = .09, *p* = .78. Collapsing of participants in this analysis was not confounding as no differences were found between Experiments (see section 3.5).

**Figure 3:**
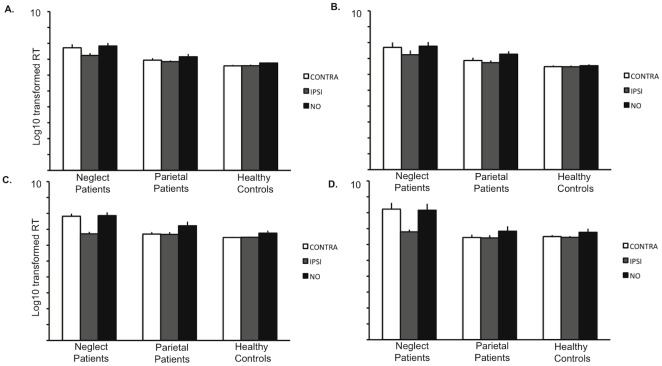
Bar graphs showing equally slow responses to neglected and absent stimuli trials in the neglect group compared to control groups for each task in both experiments. Mean reaction times (based on the log10 transformed raw data) and standard errors for each group in each condition are shown. A: Experiment 1 motor task. B: Experiment 1 verbal task. C: Experiment 2 motor task. D. Experiment 2 verbal task. CONTRA (in the neglect group) = misses of contralesional targets; CONTRA (in the control groups) = hits of contralesional/lateral targets; IPSI = hits of ipsilesional/lateral targets; NO = correct responses to target absence.

To specifically assess the fast denial theory, paired sample t-tests were conducted for every *a priori* stimulus condition pair (e.g. contralesional misses versus ipsilesional hits) in each task and experiment. This analysis failed to support fast denials of neglected information. Instead, RTs of neglected and detected stimuli were significantly different in each task in both experiments, with patients being faster when detecting the ipsilesional stimulus. Additionally, reaction times between neglected and absent stimuli trials were not significantly different, indicating equal reaction times during these conditions. This finding was consistent across both tasks in each Experiment, and group means are depicted in Figure 3. Paired t-test statistical results are presented in Table 4.

**Table 4:** Paired t-test results for logarithmic base 10 transformed reaction times in each condition-pair across both experiments in each group.

Tasks	Neglect Patients		Parietal Patient Controls		Healthy Controls	
	*t*-value	*p*-value	*t*-value	*p*-value	*t*-value	*p*-value
**Experiment 1 Verbal**						
Contra vs. Ipsi	4.650	.010**	1.821	.143	.523	.629
Contra vs. No	−.949	.396	−2.917	.043*	−1.747	.156
Ipsi vs. No	−8.510	.001**	−2.995	.040*	−1.944	.124

	Mean	St. Dev	Mean	St. Dev	Mean	St. Dev
Contra	7.697	.709	6.873	.394	6.583	.135
Ipsi	7.238	.590	6.739	.308	6.479	.169
No	7.773	.592	7.272	.408	6.548	.185
**Experiment 1 Motor**						
Contra vs. Ipsi	3.029	.039*	1.704	.164	−.865	.436
Contra vs. No	−.936	.402	−3.231	.032*	−3.299	.030*
Ipsi vs. No	−5.282	.006**	−2.711	.053	−2.688	.055

	Mean	St. Dev	Mean	St. Dev	Mean	St. Dev
Contra	7.720	.504	6.942	.279	6.583	.135
Ipsi	7.239	.308	6.852	.166	6.593	.150
No	7.833	.425	7.154	.395	6.761	.090
**Experiment 2 Verbal**						
Contra vs. Ipsi	4.324	.012**	1.233	.285	2.112	.125
Contra vs. No	0.781	.478	−1.531	.201	−1.820	.166
Ipsi vs. No	−3.990	.016**	−1.552	.196	−1.951	.146

	Mean	St. Dev	Mean	St. Dev	Mean	St. Dev
Contra	8.224	.875	6.442	.407	6.500	.182
Ipsi	6.780	.304	6.413	.390	6.451	.153
No	8.155	.913	6.838	.691	6.770	.445
**Experiment 2 Motor**						
Contra vs. Ipsi	12.071	<.001**	.499	.644	−.269	.805
Contra vs. No	−.389	.717	−3.921	.017*	−2.240	.111
Ipsi vs. No	−10.377	<.001**	−3.739	.020*	−2.092	.128

	Mean	St. Dev	Mean	St. Dev	Mean	St. Dev
Contra	7.819	.365	6.701	.293	6.488	.087
Ipsi	6.720	.274	6.685	.296	6.450	.070
No	7.860	.460	7.224	.527	6.761	.309

CONTRA (in the neglect group) = misses of contralesional targets; CONTRA (in the control groups) = hits of contralesional/lateral targets; IPSI = hits of ipsilesional/lateral targets; NO = correct response to target absence.

** Indicates p-values corrected for multiple comparisons (Bonferroni, p<.016).

* Indicates p-values that meet criteria for significance at the uncorrected (p<.05) threshold.

#### 3.2.2. Reaction Time for Control Participants

We tested for differences between stimuli and task type in both control groups using a linear mixed model. In the parietal-lesion patient group there was a significant main effect of stimulus type in both Experiments 1 and 2, *F*(2, 8.12) = 8.38, *p* = .01, *F*(2, 8.02) = 4.95, p = .04, respectively. Both experiments showed a null result for task type, *F*(1, 7.14) = .60, *p* = .47 and *F*(1, 5.70) = 1.27, *p* = .31.

Overall, results from the healthy control group were very similar to the parietal-lesion patient control group. In Experiment 1 there was a significant effect for stimulus type, *F*(2, 5.56) = 10.51, *p* = .013, but not for task type, *F*(1, 4.86) = 3.82, *p* = .11. In Experiment 2 this group showed no significant effects of stimulus type, *F*(2, 6.71) = 4.16, *p* = .07 or task type, *F*(1, 4.99) = .02, *p* = .90. See Table 4 for paired t-test comparisons for both control groups.

### 3.3. Experiment 1 Compared to Experiment 2: Participants With Neglect Respond Slower to Ipsilesional Stimuli in Experiment 1

To address the effects of stimulus duration in a visual search task we compared neglect patient reaction times in each stimulus condition between Experiments 1 and 2. Specifically, we were interested in comparing neglect patients' responses to untimed versus timed stimuli. Because several participants completed both tasks within the same experiment, we analyzed between experiment effects by task. As in previous analyses, we conducted a linear mixed effects model to account for repeated subjects between experiments in the verbal condition. No differences were found between experiments, *F*(1, 5.54) = .12, *p* = .74, and again there was a stimulus effect, *F*(2, 6.38) = 16.57, *p* = .003.

Only one person was repeated between experiments in the motor task, therefore a linear mixed effects model could not be performed. This person was removed from the analysis and a repeated measures analysis of variance (ANOVA) test was conducted using experiment and stimulus type as the between and within subjects factors. There was no difference between experiments, *F*(1, 6) = .80, *p* = .40 but there was a significant main effect for stimulus, *F*(2, 12) = 50.97, *p*<.001.

**Figure 4:**
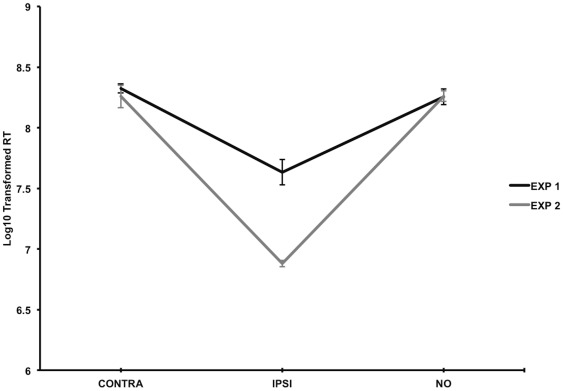
Graph showing reaction time difference in the neglect patient group between Experiments 1 and 2 when ipsilesional stimuli were detected. Data is taken from independent samples collapsed across the motor and verbal tasks. Mean reaction times (based on the log10 transformed raw data) and standard errors are shown. CONTRA = misses of contralesional targets; IPSI = hits of ipsilesional targets; NO = correct responses to target absence.

It was our *a priori* goal to examine duration effects for each stimulus condition (contra, ipsi, no) between Experiments 1 and 2 in the neglect patient group. Interestingly, results from this analysis showed that the only significant difference occurred between detected ipsilesional stimuli. Specifically, patients were significantly slower when responding to ipsilesional stimuli in Experiment 1 compared to Experiment 2 (see Figure 4). This core result was shown using independent sample t-tests after removal of repeated subjects, combining motor and verbal tasks in the same sample (*t*(7) = 3.85, *p* = .01). Also, when separating independent sample tests per motor and verbal task, the overall results of this analysis did not change in the motor task (*t*(6) = 2.92, *p* = .03). An individual test for the verbal task could not be conducted however because the sample size was too small. Reaction times were not significantly different between experiments when patients neglected contralesional stimuli or when they responded to absent stimuli trials (*t*(7) = .32, *p* = .76; *t*(7) = −.06, *p* = .96). Overall, these results consistently show a duration effect that was specific to detecting targets in the ipsilesional visual field.

## Discussion

Our goal of this study was to investigate the effects of different response modalities and stimulus durations on hemispatial visual neglect behavior and unconscious processing. Specifically, we measured reaction time and accuracy when patients with neglect responded either motorically or verbally to timed and untimed stimuli presented in a visual search task. The theory of a fast denial account was assessed with these measurements. To the best of our knowledge, comparing verbal to non-lateralized motor responses in patients with visual neglect is a novel comparison.

### 4.1. Failed to Support the Fast Denial Theory of Unconscious Processing

#### 4.1.1. Slow Responses to Neglected Stimuli

Overall, our results failed to support evidence for the behavior of unconscious processing based on the fast denial theory [15]. In fact, results from both experiments failed to show any evidence of unconscious processing when patients neglected stimuli in their contralesional visual field. Instead, the most reproducible finding across all four tasks was that all neglect patients responded to contralesional stimuli in the same manner as when the stimuli were truly absent, such that reaction times to neglected stimuli were the same as stimulus-absent trials.

Laeng et al. [16] reported a single case study of a neglect patient whose behavior also did not support evidence for the fast denial theory. Instead, their patient was faster to neglect than to detect stimuli. These fast responses were proposed to reflect pre-attentive processes during unconscious processing, and were affected by the uncertainty of stimulus presence. According to Laeng et al., the pre-attentive processes that facilitated fast neglect responses was specific to a pop-out effect of the search task, as this was not seen during a conjunction search task. Given that our results failed to replicate these findings as well, it is suspected that our study failed to elicit any form of unconscious processing because no pre-attention mechanism was initiated despite our effort to induce a pop-out effect (Experiment 2).

Attention is necessary at any level of information processing. Previous research has shown that facilitation of pre-attentive mechanisms during information processing in healthy individuals can occur, for example, by providing the context of a scene [27], grouping [28], and by providing a cue. Patients with neglect can actually increase their performance in detecting contralesional information with such facilitators [29]–[32], emphasizing that pre-attentive processing is important for contralesional information gathering (conscious or unconscious). It has been suggested that neglect results from specific attention impairments, such as the inability to orient to the contralesional space [30] or to disengage from the ipsilesional side [29].

It is very possible that our experiment design did not engage the necessary attention processes that would have resulted in overt unconscious processing behavior. It is also possible that the severity of neglect shared by patients within this group may have contributed to our findings. All patients met two out of the three criteria for assessing clinical symptoms of neglect, when in many cases only one of the assessments is needed to diagnose neglect disorder. It would therefore be expected that stronger pre-attentive cues would be needed to facilitate contralesional information gathering by patients with severe neglect, even at the subconscious level. Future studies should therefore attempt to disentangle the effects of pre-attentive mechanisms and unconscious processing in patients with varying degrees of hemispatial neglect.

#### 4.1.2. Importance of Ipsilesional Information Processing

Most experiments conducted on unilateral neglect have focused on the properties and mechanisms of when patients neglect information in their contralesional space. However, few studies have been interested in the behavior that occurs when patients are consciously aware of information in their ipsilesional space, even though it has been shown that ipsilesional information can influence neglect behavior [3]. An important finding from our study is that patients with neglect were significantly slower to respond to ipsilesional targets in Experiment 1 compared to Experiment 2. Again, the main difference between these experiments was that the stimulus remained on the computer screen in Experiment 1 but was presented for only a brief duration in Experiment 2.

Slower responses to ipsilesional targets by patients with neglect in Experiment 1 suggest that the only processing affected by stimulus duration in this study was during conscious detection of ipsilesional information. Thus, differences between reaction times of neglected and detected stimuli were not just a factor of neglect-specific behavior, but also a factor of when patients consciously detected a stimulus. This challenges the claim by Mijović-Prelec et al. [15] “…that the denied targets and the detected targets were processed in the same way…” (p. 157). In other words, our results from Experiments 1 and 2 suggest that different processes are in fact taking place when stimuli reaches awareness; and are influenced by whether or not the stimulus remains in the visual field.

#### 4.2. General Consistency Across Motor and Verbal Tasks

Overall, reaction times for the response conditions (contra, ipsi, no) between the verbal and motor tasks did not differ in Experiment 2, but were slower in the motor task in Experiment 1. After collapsing participants across experiments however, no differences were found between tasks. It could be suggested that priming a motoric system (without a lateral movement confound) may resemble the verbal system in patients with neglect. Even though this finding was against our hypothesis, the implications of this finding may be of invaluable importance to future studies. Specifically, it may be possible to use these two methods interchangeably when implementing a visual search task with this patient group. This could, therefore, increase the accessibility of patients with neglect who may be unable to make a verbal response (a common disability post stroke).

Still under debate is the prevalence of hemispatial visual neglect among patients with left hemisphere lesions. It is the typical consensus that neglect most commonly occurs after damage to the right hemisphere [1], [33], [34], although it could also occur after left-sided damage [35]–[37]. Prevalence rates of right-sided neglect may be higher than what is reported, possibly influenced by language impairments commonly seen after damage to the left hemisphere. For example, the presence of aphasia may mask the expression or diagnosis of neglect in patients with left hemispheric injury.

Integration of motor-based responses into diagnostic or experimental paradigms may help alleviate some of the methodological issues regarding evaluations of neglect patients with comorbid language impairment. It may also lead to better estimates of prevalence rates among left hemisphere/right-sided visual neglect. It is important however for future studies to further test motor and verbal responding methods and results before these assumptions can be validated.

### 4.3. Conclusions

Although our study did not elicit overt unconscious processing behavior, it cannot be definitively concluded that this type of processing did not take place. Behavioral evidence of unconscious processing has converged with neuroimaging results showing cortical activation to neglected information [34]. In one example using fMRI, a patient with comorbid visual extinction and neglect showed amygdala activity to unconsciously perceived fearful faces [38]. In a more recent study, Vuilleumier et al. [39] used fMRI to examine right and left retinotopic cortical activation in the occipital lobe when neglect patients viewed checkerboard images displayed in both hemifields. In this study, significant bi-lateral cortical activation occurred despite the failure to acknowledge these images in the neglected visual field. Therefore, future studies could benefit from the incorporation of neuroimaging techniques, which may better parse the characteristics and mechanisms behind unconscious processing in hemispatial neglect.

Collectively, our study failed to support the fast denial theory. Patients with neglect reported the same to neglected and absent stimuli, yet were affected by stimulus duration when detecting targets in the ipsilesional field. Additionally, our results suggest that patients with neglect sometimes behave similarly to visual information when they respond verbally or motorically (without a lateral confound). This finding may be invaluable for accessing patients with verbal impairment either for diagnostic or experimental purposes. Using motor and verbal responses interchangeably may also lead to better estimates of hemispatial neglect prevalence rates of those with left hemisphere damage. Given that this is a novel comparison between non-lateralized motor and verbal responding in patients with neglect, more studies need to be conducted to test this theory. Future studies also need to disentangle the effects of pre-attentive mechanisms and other factors that could affect unconscious processing along a spectrum of symptom severity. Last, incorporation of neuroimaging techniques in future studies could provide valuable information behind the mechanistic underpinnings of unconscious processing in hemispatial visual neglect.
